# Microbiota of interdental space of adolescents according to Risk of Caries: A cross-sectional study protocol

**DOI:** 10.1016/j.conctc.2019.100444

**Published:** 2019-10-18

**Authors:** Camille Inquimbert, Denis Bourgeois, Nicolas Giraudeau, Paul Tramini, Stéphane Viennot, Claude Dussart, Florence Carrouel

**Affiliations:** a Systemic Healthcare Laboratory EA4129, Faculty of Medicine Laennec, University Lyon 1, University of Lyon, Lyon, France; b Department of Public Health, Faculty of Oral Medicine, University of Montpellier, Montpellier, France; c Department of Fundamental and Clinical Biological Sciences, Faculty of Oral Medicine, University Lyon 1, University of Lyon, Lyon, France

**Keywords:** Interdental microbiota, Adolescent, Dental caries, Carious risk

## Abstract

Dental caries is a major oral disease resulting from a complex interaction between the commensal microbiota, host susceptibility (heredity, immunity, diseases, etc.) and environmental factors (diet, dental hygiene, etc.). To predict the patient's risk of new carious lesions or progression of existing lesions, the Caries Risk Assessment (CRA) takes account of clinical, biological and behavioural factors. Thus, the CRA can predict whether the patient is at high or low risk of developing caries. The practitioner can thus set up a follow-up adapted to the risk of the patient. However, although bacteria are the main etiological factor of carious lesions, the CRA does not consider bacterial quantification or just focus on the level of *S. mutans* in the saliva. As the majority of cavities are interproximal in adolescence, the aim of this trial is to identify and quantify the interdental microbiota of adolescents aged from 15 to 17 years with low or high carious risk. So, the quantification of new biomarkers associated with carious lesion could be added to the CRA to improve it.

## 1 Introduction

Caries is a major oral clinical disorder induced by a compound of microbiota whose composition varies continuously depending on the chemistry and environment of the dental site but also other more general factors such as saliva [1]. The main determinant of the evolution of the biofilm at a particular tooth site is its environment and whether the severity level of the disorder is such that it would lead to demineralization and visible changes at the site [1]. Although caries are clearly bacterial diseases, they are not infectious diseases in the classical sense because they result from a complex interaction between the commensal microbiota, host susceptibility (heredity, immunity, diseases …) and environmental factors (diet, dental hygiene …) [2].

The risk factors for caries are clinical, biological and behavioural. They predict the patient's risk of new carious lesions or progression of existing lesions [3]. By analysing all the risk and protective factors for a patient's carious disease, the clinician can assess the patient's risk for the future. This analysis procedure is called a Caries Risk Assessment (CRA) [1]. If there is an increasing interest in incorporating CRA into routine, the determining factors of caries disease are mainly dental practice, age, gender, ethnicity, oral hygiene habits, diet, education and socio-economic status [4].

The CRA for children and adolescents doesn't consider bacterial quantification, a factor highly linked to the development of caries [5]. In fact, the main factor initiating dental decay is the oral microbial dysbiosis [6]. During the carious process, the composition of the microbiota evolves. In the absence of carious lesion, the microbiota of dental enamel is principally composed of non-mutans *streptococcal* and *actinomyces* bacteria*.* Aciduric bacteria, like “low-pH” non-mutans *streptococcal* bacteria, progressively accumulate. Then, acidogenic and aciduric bacteria, like *streptococcus mutans*, *lactobacillus*, *actinomyces*, and *bifidobacterium*, become dominant [6].

Moreover, depending on the location of the dental site, the risk of developing caries varies considerably [7,8], however CRA tools do not currently use this information. The majority of carious lesions in adolescents, a period during which tooth decay is still very active [9,10], are observed on the interproximal surfaces of posterior teeth due to specific anatomical, physiological, diet-related and histological considerations [11,12]. So, the analysis of the interdental microbiota of adolescents is essential for improving our understanding of the carious process factors and for providing means to facilitate caries prediction and prevention.

This trial aims to identify as well as quantify interdental microbiota in adolescents aged from 15 to 17 years. As a contribution to increase the sensitivity of CRA, the secondary outcome is to evaluate qualitatively and quantitatively the microbiota of interdental space association according to Carious Risk.

## 2 Methods

### 2.1 Ethics statement

The protocol and design of the study named MIARC “Microbiota of Interdental space of Adolescents according to Risk of Caries”, have been endorsed by ethical and regulatory authorities and will be carried out in conformance with the Declaration of Helsinki. The Protection of Individuals Board of South-Est VI (France) approved the protocol on November 6, 2017. The National Agency for Medical and Health Product Safety registered it on February 13, 2017 (ID-RCB ref: 2017–A00425–48). The National Commission for Information Technology and Liberties gave its approval on November 6, 2017. This study was registered with ClinicalTrials.gov (identification number NCT03700840).

All participants, their parents or legal guardians will give their informed consent before participating in the study. The consent form will be composed of the following details: name and affiliation of investigator, explanations concerning the aim readily understandable by all, the course and the duration of the study, as well as the freedom to withdraw at any time, the endorsement of the ethics committee and the guarantee of confidentiality. Each participant will receive oral assessment free of charge.

### 2.2 Study design, site and population

**Fig. 1:**
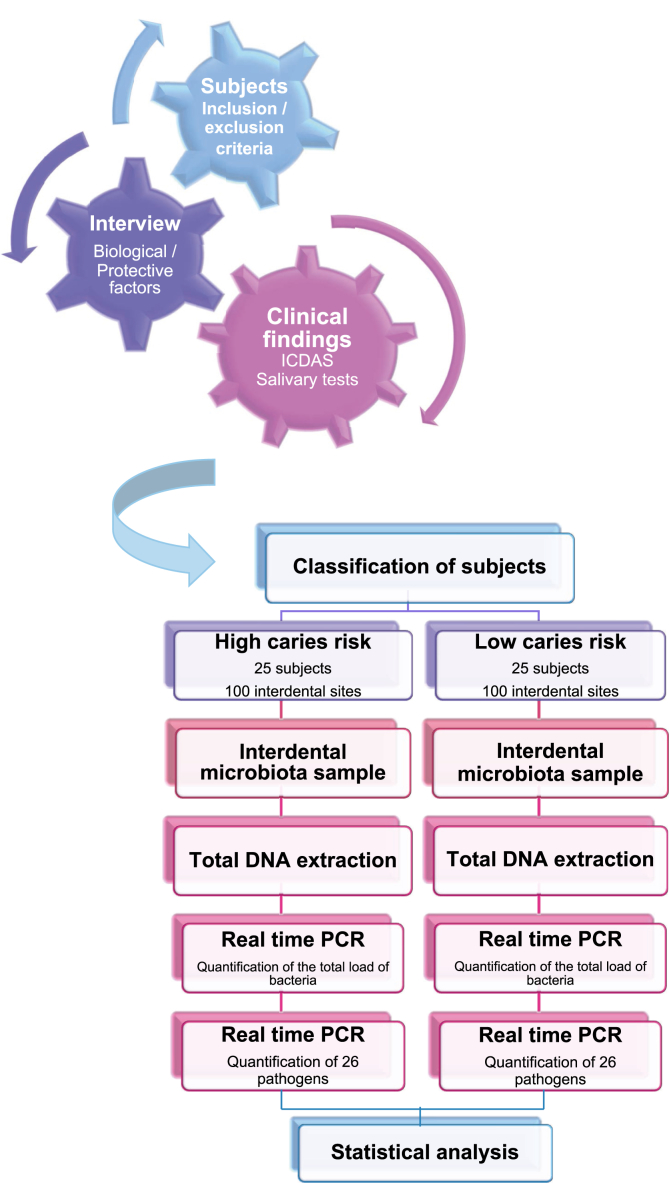
Workflow of the experiment. ICDAS: International Caries Detection and Assessment System; PCR: Polymerase Chain Reaction.

The MIARC trial is a cross-sectional observational clinical study (Fig. 1). The cross-sectional studies guidelines set out in the statement, Strengthening the Reporting of Observational Studies in Epidemiology (STROBE), will be followed [13]. Programmed for a period of 2 months in 2019, the MIARC trial will be conducted with healthy adolescents aged 15–17 years at the Dentistry Clinic Department of the Hospital of Montpellier, France. Most of the patients belong to an urban and mainly low-income population living in or around Montpellier. However, in some cases, patients come from far away to have specific care under general anaesthetic or for non-standard dental or medical treatments.

Patients will be recruited during their follow-up visit in the Public Health Department of the Oral Medicine Hospital. All potential participants (Caucasian subjects aged 15–17 years) will receive a document about the study. A toll-free number will be made available for potential participants to obtain additional information should they wish. Adolescents who agree to participate will be provided with verbal and written information about the study. They will then be interviewed at the reception area of the Public Health Department. The oral examination will be conducted at the dentistry clinic to verify the individual eligibility criteria. All patients will be contacted again to schedule an appointment if they agree.

### 2.3 Study population

Fifty Caucasian subjects will be selected from a group of first-time volunteers. The male/female ratio will be 1.0 as well as the CRA high risk/low risk ratio in the enrolled population.

The individual eligibility criteria are (i) aged 15-17 years-old, (ii) presence of teeth (15–16, 25–26, 35–36, and 45–46), (iii) having at least 22 natural teeth, (iv) good understanding of the French language, (v) one of the parents/legal guardians accepts the study's terms and conditions and signs the informed consent form, (vi) the adolescent accepts the terms and conditions of the study and signs the informed consent form. Moreover, the eligibility of the participant depends upon the presence of all his/her four premolar-molar pairs.

The clinical interdental site inclusion criteria are: (i) interdental brushes can penetrate into the four sites analysed, (ii) absence of diastemata, (iii) no interdental caries or prosthetics restorations in the teeth surrounding the interdental sites, (iv) absence of oral inflammation and bleeding on probing (BOP) after 30 s), (v) no clinical attachment loss (CAL) > 3 mm or pocket depth (PD) > 3 mm.

The criteria for exclusion are: (i) smokers, (ii) having an immune system disorder, (iii) having any other concomitant systemic illness, (iv) taking medication, (v) had a professional prophylaxis within the 4 weeks preceding the initial examination, (vi) taken antibiotics in the past 3 months, (vii) regularly using interdental brushes and/or dental floss and/or mouthwash, (viii) having a history of periodontal disease or treatment, (ix) undertaking a course of dental or orthodontic treatment, and (x) unable to answer questions or non-cooperative.

### 2.4 Repartition of subjects according to caries risk conditions

The American Academy of Pediatric Dentistry [3] sets out criteria which will be used, with certain modifications, to classify patients according to their carious risk (Table 1). The low carious risk subjects presented Caries Protective Terms, i.e., brushing daily with fluoridated toothpaste and regular dental care, Caries Risk Indicators, i.e., socio-economic status (medium, high) and snacking (low, medium) and Clinical Indicators, i.e., less than one interproximal lesion and no enamel defects nor active white spot lesions and adequate saliva flow rate. The high carious risk subjects presented Caries Protective Terms, i.e., no daily brushing with fluoridated toothpaste and/or irregular dental care and/or Caries Risk Indicators, i.e., low socio-economic status and high snacking and/or Clinical Indicators, i.e., more than one interproximal lesion and/or active white spot lesions and/or enamel defects and/or inadequate saliva flow rate.

**Table 1:** Adolescent caries risk assessment.

FACTORS	HIGH RISK	LOW RISK
Biological (interview)		
- Patient with low socioeconomic status	Yes	No
- Patient has >3 between meal sugar-containing snacks or beverages per day	Yes	No
Protective (interview)		
- Patient brushes teeth daily with fluoridated toothpaste	No	Yes
- Patient has regular dental care	No	Yes
Clinical Findings (International Caries Detection and Assessment System (ICDAS) and salivary tests)		
ICDAS		
- Patient has >1 interproximal lesion	Yes	No
- Patient has active white spot lesions or enamel defects	Yes	No
Salivary tests (hydration, salivary consistency, resting saliva pH, stimulated saliva flow, stimulated saliva pH and saliva buffering capacity)		
- Patient has high salivary risk	Yes	No

### 2.5 Clinical examination

One trained and calibrated dentist who is experienced with the clinical indices will perform the clinical examination.

#### 2.5.1 Dental examination

In reference to the International Caries Detection and Assessment System (ICDAS) criteria [14], adolescents will receive a clinical oral examination. After removing the dental plaque with a toothbrush or a professional prophylaxis, drying with gauze or air (5 s), the teeth will be observed under light (Orascoptic Endeavor headlamp system) without magnification and, a ball-end probe (WHO probe) could be used to determine the ICDAS score. This score will permit to determine the presence or absence of carious lesion (code 0: absence of carious lesion, code 1 to 6: carious lesion at different stages) [14]. There will be no radiography carried out. Adolescents will be informed of any conditions requiring treatment.

#### 2.5.2 Salivary tests

This aims at investigating hydration, salivary consistency, resting saliva pH, stimulated saliva flow, stimulated saliva pH and saliva buffering capacity. All tests will be performed according to the instructions of the manufacturer (Saliva-Check Buffer test, GC France SAS, Sucy-en-Brie, France). All patients were refrained from eating, drinking, smoking, and performing oral hygiene procedures for 2 h before saliva collection. The tests will realized 30min after awakening at least, between 9:00 a.m. and 11:00 a.m [[15], [16], [17]]. The resting saliva consistency will be classified as sticky frothy or frothy bubbly or watery clear by observing the saliva in the floor of the mouth. The resting flow saliva will be evaluated by measuring the time to see new saliva droplets after drying the lip labial mucosa with a gauze (>60 s: low resting flow rate, 30–60 s: normal resting flow rate, < 30 s: high resting flow rate). The resting saliva pH will be measured with a pH strip (pH 5.0–5.8: highly acidic saliva, pH 6.0–6.8: moderately acidic saliva, pH 6.8–7.8: healthy saliva). The stimulated salivary flow rate will be analysed as the volume of saliva collected while the patient chewed a paraffin pellet for 5 min (<3.5 ml: very low, 3.5–5 ml: low, > 5 ml: normal). The salivary buffering capacity will be evaluated by depositing stimulated saliva on a test strip containing three different acid challenges.

### 2.6 Interdental microbiota sample

Four interdental sites (15–16, 25–26, 35–36, and 45–46), free from caries (ICDAS code 0), will be assessed for each subject. The adapted interdental brush (Curaden, Kriens, Switzerland) will be determined by using a Curaprox IAP calibration probe (Curaden) [18]. Then, the isolation of each tooth selected for sampling will be performed by using sterile dental cotton rolls and a calibrated, sterile interdental brushes (IDB) will serve to remove the interdental biofilm. The IBDs containing the individual samples will be stored in sterile tubes at 4 °C for later laboratory treatment [19,20].

### 2.7 Microbiological analysis

Quantification of the total number of bacteria and 26 pathogens will be performed by real-time PCR. The pathogens selected periodontal or carious bacteria: *Actinomyces odontolyticus, Aggregatibacter actinomycetemcomitans, Bifidobacterium dentium, Campylobacter rectus, Campylobacter gracilis, Capnocytophaga ochracea, Clostridium cluster IV, Clostridium cluster XIV, Eikenella corrodens, Fusobacterium nucleatum, Lactobacillus* spp.*, Parvimonas micra, Porphyromonas gingivalis, Prevotella intermedia, Prevotella nigrescens, Rothia dentocariosa, Scardovia wiggsiae, Streptococcus cristatus, Streptococcus mitis, Streptococcus mutans, Streptococcus salivarius, Streptococcus sanguinis, Streptococcus sobrinus, Tannerella forsythia, Treponema denticola,* and *Veillonella parvula.* Simplex quantitative real-time PCR assays will be performed in a volume of 10 μL (2 μL of DNA extract, 1 × SYBR® Premix Ex TaqTM Tli RNaseH Plus and 1 μM of each primer) (TaKaRa, Shiga, Japan).

### 2.8 Statistical analysis

#### 2.8.1 Sample size

With an alpha error of 5% (2-sided test), a power of 80%, an intraclass correlation coefficient of 0.8 and a mean difference of bacteria counts between the two caries risk groups of 1,300,000, a total of 200 sites, meaning 50 subjects, needs to be selected.

#### 2.8.2 Analysis

Three main steps will be realized: preparation of descriptive summaries, modelling using a mixed (linear) model and correlating bacterial abundances. A mixed linear model for the log-count abundance of each species at a measured site will be used to test for potential effects of gender, interdental space and the location of each site. Two categorical variables (site location and gender), and one numerical variable (interdental space) will be modelled as fixed effects and one categorical variable will be modelled as a random effect (subject). This random effect will be included to model correlation between the four interdental sites of a given study participant. In the regression, each coefficient will be tested according to the null hypothesis which indicates that the coefficient is zero when a likelihood-ratio test is applied. The p-values less than 0.001, 0.05, and 0.01 will be respectively considered strong, medium and low evidence with respect to the null hypothesis. To perform the correlation analysis and avoid over-estimating the inter-site correlation, the residuals of the model described above will be used. The trees associated with the correlation plot will be generated by hierarchical cluster analysis with complete linkage. Other multivariate analyses, such as factor analysis, will be used to graphically check the correlations between bacteria quantification and all potential caries risk factors. A multilevel logistic regression analysis will finally be performed to test which oral characteristics are significantly linked with the two caries risk groups. The linear mixed effects modelling package, lme4 [21] in R environment [22] will be used to generate all statistical analyses and associated plots.

## 3 Discussion

To our knowledge, the MIARC trial is the first cross-sectional clinical study which analyses the interdental microbiota according to the caries risk factor**s** of adolescents aged 15–17 years.

The sampling of site-specific microbiota for analysing the molecular properties of caries needs to reflect the localised nature of this clinical disorder. A previous study has demonstrated that the proximal surfaces were the most exposed surfaces, after the occlusal surfaces to carious lesions [23]. Indeed, the risk of interproximal carious lesions is important among the high-risk adolescents, mainly due to the lack of compliance with caries-prevention programs in this group [24]. In children aged 12 years, the prevalence of interproximal caries is 39% and increases to 72% at 20–21 years [25]. Enamel lesions on the approximal surfaces among 16-year-olds account for more than 80% of all caries lesions on these surfaces, no matter whether the caries prevalence in the population is high or low [26].

Whilst no standard benchmark or criteria for evaluating a CRA's quality exists, a universal rating of the clinical examination from ‘low to high’ will be used in this study for testing the criterion validity of carious risk. The classification criteria for CRA systems were determined by combining scientific evidence and expert opinion. From these CRAs, practitioners analyse the various clinical and social factors of a patient and can thus assign a carious risk status [27]. Our study classified patients into two risk categories: low carious risk and high carious risk. There is no necessity for further preventive professional treatment for the low risk patients and they should be offered an extended follow-up [28]. For high-risk patients, preventive actions must be taken to reduce the incidence and severity of future carious lesions [28]. This individual scheduling of preventive and follow-up activity better appropriates the use of dental resources and lowers dental costs for certain individuals [28].

In some CRA classifications, the quantification of *S. mutans* in the saliva is considered [3]. However, the results concerning the link between *S. mutans* and the development of dental caries is not clear. Some studies demonstrated a real association between *S. mutans* and the carious lesion whereas others revealed no clear association [29]. Moreover, the amount of *S. mutans* needed to initiate the carious lesion varies according to the study [30]. Recent studies revealed that the decrease of the taxonomy and the presence of key pathogens are signals preceding the carious disease [31,32]. The issue of one pathogen could be integrated with the interdental microbiota taxonomy variation. Thus, to use *S. mutans* or other bacteria as a biomarker in the CRA classification, it is necessary to identify specific biomarkers associated with carious lesions and to normalize their quantification. For this, the MIARC study had the advantage of an extensive sample of interproximal sites. Moreover, although the list is not exhaustive, the bacteria identified by the real-time PCR technique correspond mainly to those identified in the current literature as being potentially at cariogenic risk.

As we will focus on interdental microbiota, results should be more significant and correlated to the carious risk. The real-time PCR method presents the advantage of quantifying bacteria specifically. Furthermore, comparatively speaking, this method is straightforward, economical and reliable [33].

Recent progress achieved in understanding the “systems biology” of dental caries has broadened the research beyond individual factors (e.g., fluoride content, salivary pH, or measuring quantities of bacterial species) and has established a platform for the advancement of innovative approaches in caries diagnosis and management [34]. This advancement will certainly be facilitated by the development of innovative applications to monitor biomarkers and manifestations of disease as they occur.

Thus, the MIARC trial allows one to evaluate if carious pathogens are present in the interdental space of adolescents and to determine if the interdental microbiota of adolescents with a high carious risk is different from the one of adolescents with a low carious risk. This should allow one to determine a specific biomarker of each carious risk. This quantification of determined bacteria could be added to CRA to improve them. This study should help optimize recommendations in terms of oral prevention in this at-risk age group. Furthermore, the expected results will provide new information on the association between the level of carious risk, measurement of the state of oral hygiene, clinical signs of gingival inflammation, diameter of the interdental spaces and interdental microbiota composition.

In conclusion, the MIARC study is a first step before further studies, ideally longitudinal clinical studies encompassing large populations which would identify the panel of bacteria as prospective oral health markers. With this new data, oral health professionals will be able to collect and analyse interdental microbiota to prevent the risk of new caries.

## Funding

This research received no specific grant from any funding agency in the public, commercial or not-for-profit sectors.

## Declaration of competing interest

All other authors declare that they have no competing interests.
